# The TERT promoter mutation incidence is modified by germline TERT rs2736098 and rs2736100 polymorphisms in hepatocellular carcinoma

**DOI:** 10.18632/oncotarget.15498

**Published:** 2017-02-18

**Authors:** Xiaotian Yuan, Guanghui Cheng, Jingya Yu, Shunzhen Zheng, Chao Sun, Qing Sun, Kailin Li, Zhaomin Lin, Tiantian Liu, Ping Li, Yiteng Xu, Feng Kong, Magnus Bjorkholm, Dawei Xu

**Affiliations:** ^1^ Department of Medicine, Division of Hematology and Center for Molecular Medicine, Karolinska Institutet and Karolinska University Hospital Solna, Stockholm, Sweden; ^2^ Central Research Laboratory, the Second Hospital of Shandong University, Jinan, PR China; ^3^ Department of OrganTransplantation and Hepatobiliary Surgery, Shandong Provincial Hospital Affiliated to Shandong University, Jinan, PR China; ^4^ Department of Pathology, Shandong University School of Medicine, Jinan, PR China; ^5^ School of Nursing, Shandong University, Jinan, PR China

**Keywords:** HCC, promoter mutations, rs2736098, rs2736100, SNP

## Abstract

Telomerase activation via induction of the catalytic component telomerase reverse transcriptase (*TERT*) plays essential roles in malignant transformation. TERT promoter-activating mutations were recently identified as a novel mechanism to activate telomerase in hepatocellular carcinoma (HCC) and many other malignancies. In addition, single nucleotide polymorphisms (SNPs) in the TERT rs2736098 and rs2736100 are significantly associated with cancer susceptibility. It is currently unclear whether different germline TERT variants modify TERT promoter mutations. Here we analyzed the TERT promoter status and genotyped the TERT SNPs at rs2736098 and rs2736100 in patients with HCC. Thirty percent of HCCs harbored TERT promoter mutations and there was a significant difference in rs2736098 and rs2736100 genotypes between wt and mutant TERT promoter-bearing HCC tumors (*P* = 0.007 and 0.018, respectively). For rs2736100, the cancer risk genotype CC was significantly associated with a reduced incidence of TERT promoter mutations compared to AA + AC variants [Odds ratio (OR): 0.181, 95% Confidence interval (CI): 0.0543–0.601, *P* = 0.004]. The rs2736098_CT genotype was significantly associated with the TERT promoter mutation-positive tumors compared to the TT genotype (OR: 5.391, 95% CI: 1.234–23.553, *P* = 0.025). These differences in genotype distribution did not differ between patients with a wt TERT promoter and controls. The presence of TERT promoter mutations was not associated with clinico-pathological variables. Taken together, the germline TERT genetic background may significantly affect the onset of TERT promoter mutations in HCCs, which provides a better understanding of HCC-related TERT promoter mutations and telomerase regulation in cancer.

## INTRODUCTION

Hepatocellular carcinoma (HCC) is the dominant primary liver cancer with an estimated number of 466,100 new cases and 422,100 deaths, occurring in China for 2015, which is almost the half of all cases diagnosed globally [1]. During recent last decades, significant advances have been made in the understanding of HCC [2, 3]. Chronic hepatitis B or C infection, alcohol abuse, male gender and cigarette smoking have been identified as important risk factors to contribute to HCC development [1–3]. Nevertheless, the etiology and pathogenesis of HCC remains incompletely elucidated [1]. Clinically, early diagnosis and intervention is the key to patient cures or long-term survival. However, most HCC patients are diagnosed at an advanced stage, and invasive diseases or distant metastasis accounts for the majority of mortalities due to limited treatment choices [1]. Therefore, increased understanding of the HCC pathogenesis, early detection, improved diagnostic and prognostic markers, novel preventive strategies and identification of new therapeutic targets are urgently needed.

Unlimited proliferation is a key hallmark of cancer cells [4], and activation of telomerase is required to achieve it in the vast majority of cancer types including HCC [5–7]. Telomerase is a ribonucleoprotein enzyme with its catalytic unit telomerase reverse transcriptase (TERT) as rate-limiting factor, and in general silent in normal human differentiated cells due to the transcriptional repression of the *TERT* gene [5, 6]. In contrast, most HCCs and other malignancies constitutively express TERT and telomerase activity [5, 8]. The mechanism underlying cancer-specific telomerase activation/TERT expression has been extensively studied, and recent findings showed that HCC tumors frequently bear activating mutations in the TERT proximal promoter (−124 and −146 bp from the ATG, so-called C228T and C250T, respectively) [2, 8–20]. These mutations promote the TERT gene transcription, thereby activating telomerase [2, 8–16]. However, TERT promoter mutations occur in up to 50% of HCCs and the mutation frequency varies significantly with tumor types [8]. It remains unclear how and why such variations take place.

The central role of TERT in oncogenesis has also promoted studies of cancer susceptibility and association with single nucleotide polymorphisms (SNPs) in the *TERT* locus, and the accumulated evidence indeed suggests the association between cancer risk and *TERT* SNPs [17, 21–36]. Among all the TERT SNPs, rs2736100 at intron 2 and rs2736098 at exon 2 are most studied. The rs2736100 CC genotype was reported to confer an increased risk of different cancer types [21]. Mechanistically, the rs2736100 CC may up-regulate TERT expression through which its oncogenic effect is exerted [34]. The relationship between rs2736098 variants and cancer risk is complicated and risk alleles vary in different types of cancer [25, 31]. Moreover, it remains poorly understood whether and how rs2736098 variants affect TERT activity [37]. Nevertheless, the rs2736098 A allele was shown to significantly increase HCC risk.

Both TERT promoter mutations and TERT SNPs play important roles in oncogenesis, but it is unclear whether they interact or associate with each other. To address this issue, we analyzed HCC tumors for TERT promoter mutations and relationship with rs2736100 and rs2736098 variants.

## RESULTS

### TERT promoter mutations and relation to clinico-pathological variables in HCCs

**Figure 1:**
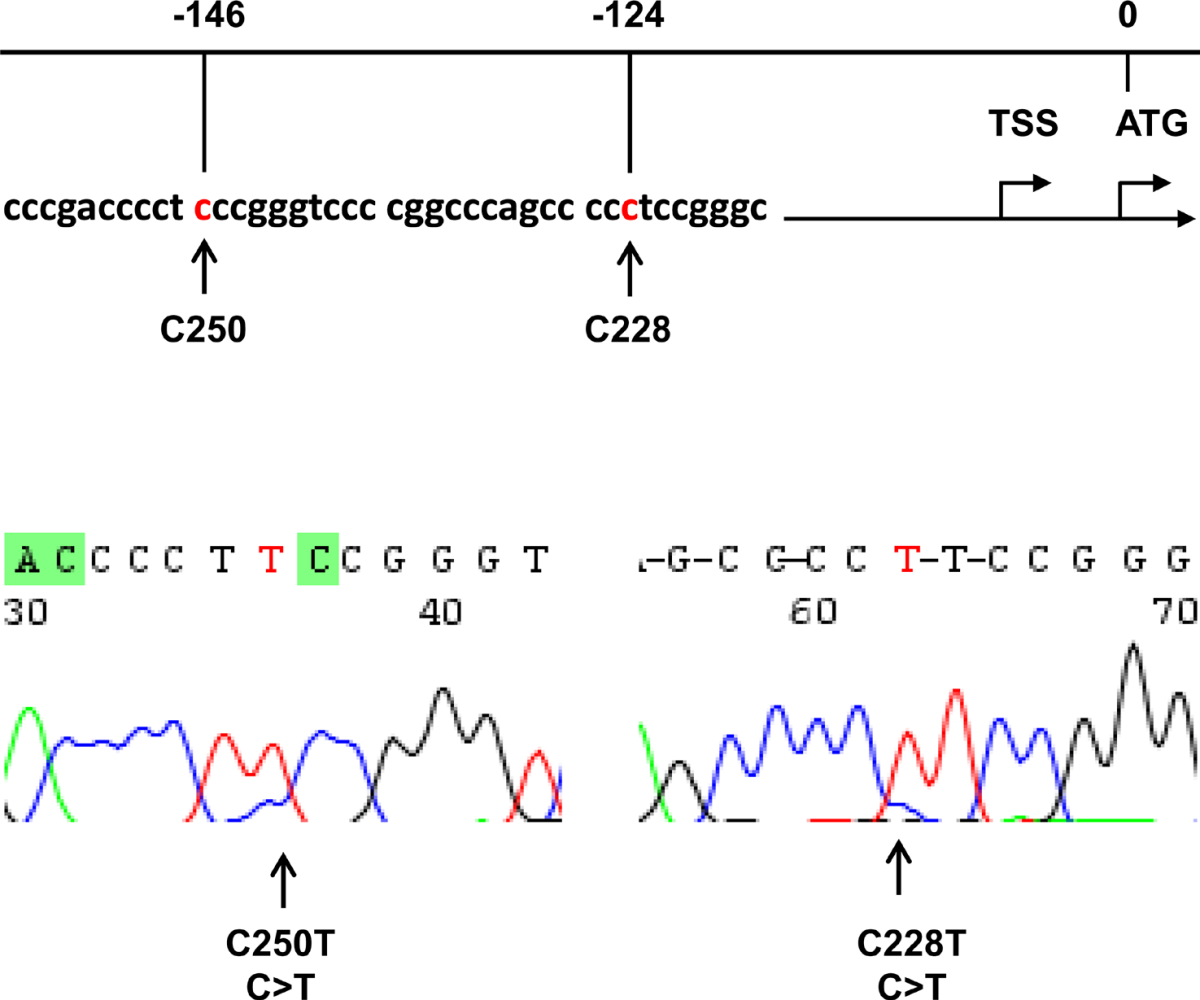
Identification of *TERT* promoter mutations in hepatocellular carcinoma (HCC) Top: Location of C228T and C250T (in red) in the *TERT* core promoter. TSS: Transcription start site. Bottom: Sequencing chromatographs of the *TERT* promoter locus in genomic tumor DNA from two HCC patients obtained by Sanger sequencing.

The TERT promoter was sequenced in DNA from 200 HCC patients and 190 of them were evaluable. Fifty-seven of 190 HCC tumors (30%) harbored TERT promoter mutations, among which 50 were C228T while 7 C250T (Figure 1). Clinic-pathological variables were compared between patients with and without TERT promoter mutations in their tumors, and there were no differences in age, sex, HBV infection, liver cirrhosis, α-fetoprotein levels, tumor sizes, differentiation status and metastasis (Table 1).

**Table 1:** TERT promoter mutations with clinical characteristics in HCC patients

*TERT* promoter mutation			
Variable informative cases (*N* = 190 )	Mutated (*N* = 57 )	wild-type (*n* = 133 )	*P*-value
*Age at diagnosis* (*n* = )	56	128	0.1905
Mean years	54.71	52.63	
Median (range) years	55.5 (32–75)	51 (25–76)	
*Gender* (*n* = )	56	128	0.898
Female	8	19	
Male	48	109	
*HBV infection* (*n* = )	55	129	0.105
Yes	50	103	
No	5	26	
*Cirrhosis* (*n* = )	57	130	0.394
Yes	30	58	
No	27	72	
*α-fetoprotein (ng/ml)*	54	120	0.927
< 200	38	82	
≥ 200	16	38	
*Tumor size* (*n* = )	56	121	0.328
< 5 cm	32	58	
> 5 cm	24	63	
*Differentiation*	55	123	0.609
Well or moderate	37	89	
*Poor*	18	34	
*CTNNB1 (n = ) or TERT (n = )*			
mutated	6	11	0.535
wt	13	38	
*Metastases* (*n* = )	56	129	0.67
Yes	1	5	
No	55	124	

HCC, Hepatocellular carcinoma; HBV, Hepatitis B virus.

### TERT rs2736098 and rs2736100 variants and relation to HCC risk

Since SNPs at rs2736098 and rs2736100 have been shown to be associated with increased cancer risk, we determined whether these genetic variants have any effects on HCC susceptibility by comparing their genotype distributions with healthy controls. The genotyping data were available in 240 healthy controls and 231 HCC patients for rs2736098, while in 237 healthy controls and 201 HCC patients for rs2736100, respectively. Table 2 summarizes the genotyping results and both patients and controls exhibited comparable genotype and allele frequencies of rs2736098. However, the rs2736100_CC genotype was significantly lower in HCC patients than in healthy controls (OR: 0.544, 95% CI: 0.320–0.925, *P* = 0.034) (Table 2).

**Table 2:** TERT rs2736098 and 2736100 genotyping in healthy adults and HCC patients

	HA	HCC	Odds ratio (95% CI)	*P* value
**Genotype**				
rs2736098 (N)	240 (100%)	231 (100%)		
TT	31 (12.9)	19 (8.2)	1.0 (ref.)	
CT	115 (47.9)	127 (55.0)	1.802 (0.965–3.364)	0.088
CC	94 (39.2)	85 (36.8)	1.475 (0.7763–2.804)	0.303
CT + CC	209 (87.1)	212 (91.8)	1.655 (0.906–3.023)	0.133
CC	94 (39.2)	85 (36.8)	1.0 (ref.)	
TT + CT	146 (60.8)	146 (63.2)	1.106 (0.762–1.605)	0.664
rs2736100 (N)	237 (100%)	201 (100%)		
AA	69 (29.1)	74 (36.8)	1.0 (ref.)	
AC	108 (45.6)	92 (45.8)	0.794 (0.517–1.221)	0.347
CC	60 (25.3)	35 (17.4)	0.544 (0.320–0.925)	**0.033**
AC + CC	168 (74.7)	127 (63.2)	0.705 (0.472–1.053)	0.107
CC	60 (25.3)	35 (17.4)	1.0 (ref.)	
AA + AC	177 (73.7)	166 (82.6)	1.608 (1.007–2.566)	0.060

HCC, Hepatocellular carcinoma; CI, Confidence interval.

### No association between TERT promoter mutations and CTNNB1 mutations in HCC tumors

The *CTNNB1* gene mutation is frequent in HCCs and recent studies showed its close association with TERT promoter mutations. We thus further sequenced the *CTNNB1* exon 3 for the hotspot mutations in 81 HCC tumors [13, 14]. The sequencing data were evaluable in 70 of them and the mutation found in 17 tumors (24.3%). The TERT promoter sequencing was successful in 68 of these tumors. There were 6 (35.3%) and 13 (23.5%) with mutant TERT promoters in 17 *CTNNB1* mutation-positive and 51 mutation-negative HCCs, respectively, this difference being non-significant (*P* = 0.535) (Table 1).

### The TERT promoter mutation and association with TERT genetic variants in HCCs

As TERT SNPs at rs2736098 and rs2736100 were observed to regulate TERT expression and telomerase activity [31, 34, 37], we sought to determine the relationship between the TERT promoter mutation and SNPs at these two loci. HCC patients with tumors bearing mutant TERT promoter exhibited remarkably lower frequencies of rs2736098_TT and rs2736100_CC genotypes compared with those of healthy controls (Table 3) (mutant cases vs controls: 3.6% vs 12.9% and 5.8 vs 25.3% for rs2736098_TT and rs2736100_CC, respectively). Compared to rs2736098_TT cases, HCC patients with rs2736098_CT genotype had 5.39-fold increase in TERT promoter mutation-positive tumors (OR: 95% CI: 1.234 – 23.554, *P* = 0.025). Patients carrying rs2736100_AA and AC variants exhibited 5.5-fold increase in having TERT promoter-mutant tumors (OR: 0.181, 95% CI: 0.054 – 0.601, *P* = 0.004). Similar differences in the genotype of rs2736098 and rs2736100 were also seen between patients with wt and mutant TERT promoters (mt vs wt: *P* = 0.007 and 0.018 for rs2736098 and rs2736100, respectively) (Table 4). In contrast, we did not observe significant differences in both rs2736098 and rs2736100 genotype distributions between healthy controls and HCC patients carrying a wt TERT promoter (Table 3).

**Table 3:** TERT promoter mutations and association with rs2736100 and rs2736098 in HCC patients

Genotype	Cases	Healthy controls	Odds ratio (95% CI)	*P*
**rs2736098**				
wt TERT promoter vs controls	128 (100%)	240 (100%)		
TT	13 (10.1)	31 (12.9 )	1.0 (Ref.)	
CT	61 (47.7)	115 (47.9 )	1.265 (0.617–2.594)	0.643
CC	54 (42.2)	94 (39.2 )	1.370 (0.661–2.840)	0.504
CT + CC	115 (89.9)	209 (87.1)	1.310 (0.660–2.607)	0.543
mt TERT promoter vs controls	55 (100%)	240 (100%)		
TT	2 (3.6)	31 (12.9 )	1.0 (Ref.)	
CT	40 (72.7)	115 (47.9 )	5.391 (1.234–23.553)	**0.025**
CC	13 (23.7)	94 (39.2)	2.144 (0.458–10.030)	0.505
CT + CC	53 (96.4)	209 (87.1)	3.931 (0.912–16.948)	0.083
**rs2736100**				
wt TERT promoter vs controls	114 (100%)	237 (100%)		
AA	38 (33.3)	69 (29.1)	1.0 (Ref.)	
CA	52 (45.6)	108 (45.6)	1.141 (0.683–1.916)	0.705
CC	24 (21.1)	60 (25.3)	0.726 (0.392–1.346)	0.389
AA + AC	90 (78.9)	177 (74.7)	1.0 (Ref.)	
CC	24 (21.1)	60 (25.3)	0.787 ( 0.460–1.346)	0.457
mt TERT promoter vs controls	52 (100%)	237 (100%)		
AA	15 (28.8)	69 (29.1)	1.0 (Ref.)	
CA	34 (65.4)	108 (45.6)	1.448 (0.735–2.854)	0.389
CC	3 (5.8)	60 (25.3)	0.230 (0.0635–0.833)	**0.032**
AA + AC	49 (94.2)	177 (74.7)	1.0 (Ref.)	
CC	3 (5.8)	60 (25.3)	0.181 (0.0543–0.601)	**0.004**

HCC, Hepatocellular carcinoma; CI, confidence interval.

**Table 4:** rs2736098 and rs2736100 genotype frequency in HCC patients bearing wt and mutant TERT promoter in tumors

	wt	mutant	*P* value
rs2736098	128 (100%)	55 (100%)	
TT	13 (10.1)	2 (3.6)	
CT	61 (47.7)	40 (72.7)	
CC	54 (42.2)	13 (23.7)	**0.007**
rs2736100	114 (100%)	52 (100%)	
AA	38 (33.3)	15 (28.8)	
CA	52 (45.6)	34 (65.4)	
CC	24 (21.1)	3 (5.8)	**0.018**

HCC, hepatocellular carcinoma.

**Figure 2:**
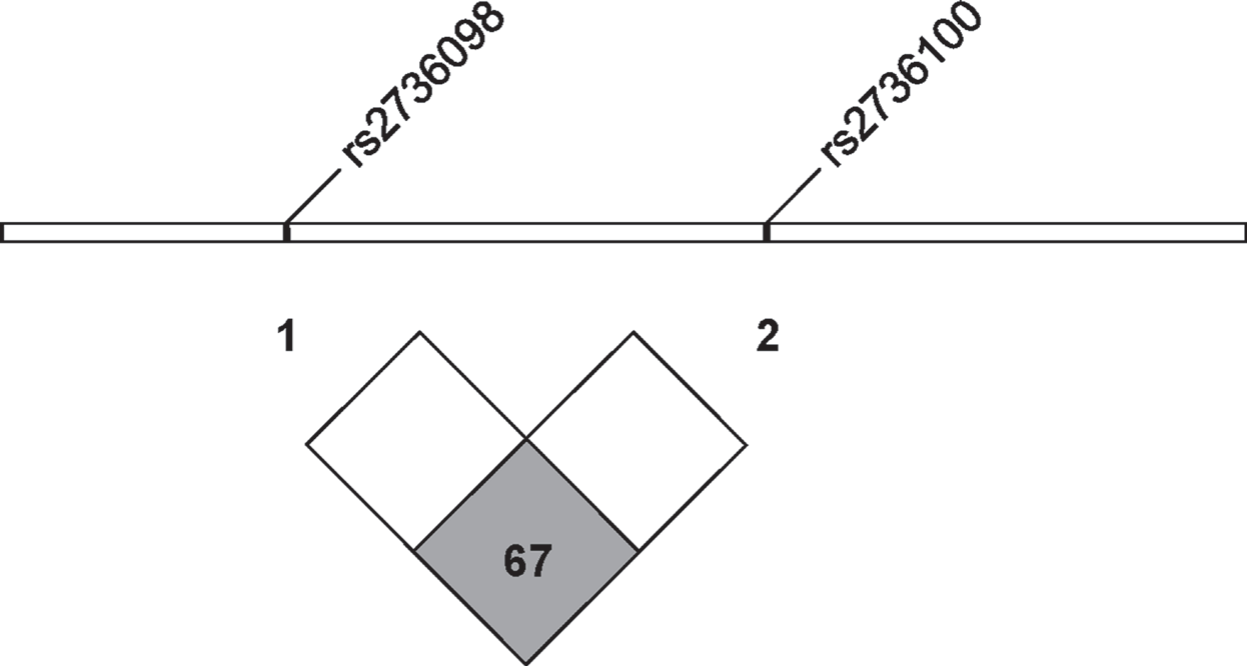
The linkage disequilibrium analysis of rs2736098 and rs2736100 in the Han Chinese population D′ = 0.67 (< 0.8 as non-significantly associated) and *r*^2^ = 0.41. (From http://www.ensembl.org).

In addition, we further performed the linkage disequilibrium analysis of rs2736098 and rs2736100 in the Han Chinese population (Figure 2). LD or D’ and r^2^ values are 0.67 and 0.41, respectively, which indicates a non-significant association between these two SNPs (< 0.8).

## DISCUSSION

Multiple oncogenic signalings contribute to the aberrant TERT induction and telomerase activation, an essential step towards malignant transformation, and the recent identification of cancer-specific hotspot mutations in the TERT proximal promoter reveals a novel mechanism behind telomerase activation [8]. It has been documented that the frequency of TERT promoter mutations varies significantly from cancer to cancer, but the underlying mechanism is unclear. In the present study, we determined TERT promoter mutations and *TERT* gene SNPs in HCC patients, and our results show a negative association between patients having TERT promoter mutation-positive tumors and rs2736098_TT and rs2736100_CC genotypes.

TERT promoter mutations, thus identified in multiple-types of human malignancies including HCCs, function as oncogenic drivers due to their stimulatory effects on TERT transcription and telomerase activation [8, 38, 39]. Both C228T and C250T mutations create extra *de novo* ETS binding motifs that are selectively bound by the ETS family transcription factor GABPA [39]. The GABPA binding leads to opened chromatin at the TERT proximal promoter, thereby aberrantly activating TERT transcription and telomerase [39]. Consistently, higher levels of TERT mRNA and telomerase activity were frequently observed in tumors carrying TERT promoter mutations compared with those having a wt promoter [8, 18, 40, 41]. On the other hand, the rs2736100_CC genotype has also been shown to promote TERT transcription and to maintain telomere length much more strongly than its AA and AC variants [17, 34]; whereas tumors with shorter telomere are prone to undergo TERT promoter mutations [42]. From this standpoint of view, HCC tumors derived from patients with rs2736100_AA or AC genotype are more genetically stressed for telomerase activation than those bearing the CC variant. It is thus conceivable that TERT promoter mutations occur predominantly in HCC patients with rs2736100_AA and AC genotypes, as observed in the present study.

We also found a significantly lower frequency of the rs2736098_TT genotype in HCCs with a TERT promoter mutation, which was similar to that of the rs2736100_CC variant. However, in contrast to the rs2736100_CC genotype that up-regulates TERT transcription, rs2736098_TT has been shown to be associated with lower TERT expression and shorter telomere length [25, 31, 43, 44]. Therefore, the reduced rs2736098_TT variant in mutant TERT promoter-bearing HCCs cannot simply be explained by its effect on TERT expression. Of note, it should also be pointed out that both rs2736100_CC and rs2736098_TT genotypes increase cancer risk, despite their opposite impacts on TERT transcription and telomere length regulation. Nevertheless, patients with TERT promoter mutation-positive HCCs have lower frequencies of cancer susceptibility genotypes rs2736098_TT and rs2736100_CC.

Up to 50% of HCCs were previously reported to have TERT promoter mutations, and those studies showed an association of the mutation with certain clinico-pathological variables including age, sex, HBV or HCV infection, α-fetoprotein, tumor sizes/numbers, differentiation status and metastasis [2, 9–16]. However, we did not find that the presence of TERT promoter mutations was associated with any of the above variables in this cohort of HCC patients.

Co-occurrence of TERT promoter mutations together with activating mutation of oncogenic drivers that facilitate cellular replication is frequent. The *fibroblast growth factor receptor 3* (*FGFR3*) gene mutation is highly prevalent in urothelial carcinomas, and the mutant FGFR3 promotes malignant development by over-stimulating the RAS-mitogen-activated protein kinase and phosphatidylinositide-3 kinase–AKT pathway [45, 46]. TERT promoter mutations are shown to be tightly associated with the presence of *FGFR3* mutations in urothelial carcinomas (UC) [45]. In HCCs, the *CTNNB1* gene, encoding β-catenin, is frequently mutated and its mutant version plays similarly important parts in enhancing HCC cell proliferation [3]. A few of studies demonstrated that the presence of the TERT promoter and *CTNNB1* mutations was correlated with each other in HCCs [12–14]. However, our results show that these two genetic events were independent of each other. Likely, different genetic susceptibility and/or environment exposure may contribute to different mutation profiles. For instance, we found that the *FGFR3* mutation was rare in Chinese patients with UC, despite its high incidence in those UC patients from Western countries [45, 47, 48].

The rs2736098_TT genotype was previously found to be associated with HCC risk in a Chinese population by Zhang et al. [25], however, we failed to demonstrate such an association. Of note, those authors showed the association of the rs2736098_TT variant only with HBV infection-negative HCC [25]. Because our cohort of HCC patients was predominantly HBV-infected, we were unable to do this same analysis. Further investigations by recruiting larger cohorts of HBV-negative HCC are apparently required to solve this discrepancy. Intriguingly, the vast majority of studies have shown rs2736100_CC genotype as a cancer risk variant [17, 21], while our present findings suggest its association with a lower risk of HCC. However, this result was obtained from relatively small cohorts of subjects (< 300), which calls for further evaluations on more healthy individuals and HCC patients for validation.

## MATERIALS AND METHODS

### Study population

Two hundred and forty-five patients with newly diagnosed histologically confirmed HCC were recruited from Shandong University Second Hospital and Shandong Provincial Hospital (Table 1). Adult sex-matched healthy individuals (*N* = 257) served as controls. Age (mean ± SD) for cases and controls was 45 ± 16 yrs and 54 ± 10 yrs, respectively. The ethnic background of both cases and controls was Han Chinese from the Shandong area. Tumor specimens and/or blood samples were obtained from all the participants after informed content. The study was approved by the Shandong University Second Hospital Ethics Committee. All experiments were performed in accordance with relevant guidelines and regulations.

### DNA extraction and Sanger sequencing of the TERT promoter and CTNNB1 gene

Genomic DNA was extracted using QIAGEN DNA extraction kits. DNA derived from HCC tumors was analyzed for the TERT promoter and *CTNNB1* gene mutations using Sanger sequencing. The two mutations defined as C228T and C250T in the TERT core promoter correspond to positions 124 and 146 bp upstream of the ATG site [38, 49] (Figure 1). The primer pair for the TERT promoter sequencing was previously described [40, 42]: 5′-CAC CCG TCC TGC CCC TTC ACC TT-3′ (forward) and 5′-GGC TTC CCA CGT GCG CAG CAG GA-3′ (reverse). The *CTNNB1* gene hotspot mutations occur in exon 3 and we chose the following primers for sequencing analysis of this region: 5′-GGG TAT TTG AAG TAT ACC ATA C-3′ (forward) and 5′-TGG TCC TCG TCA TTT AGC AG-3′ (reverse). All the mutations were verified by sequencing from both directions.

### The TERT rs2736100 (AC) and rs2736098 (TC) genotyping

The *TERT* rs2736100 (AC) and rs2736098 (TC) genotyping was carried out using pre-designed TaqMan SNP genotyping assay kits on an ABI PRISM 7900 HT Sequence Detection System (Applied Biosystems), as described [17, 24, 50]. Both positive and negative controls were included in all assays and the running condition was as followed: 95°C for 10 min, followed by 40 cycles of 92°C for 15 sec and 60°C for 1 min.

### Statistical analyses

Sex was compared between patients and controls using Chi-square (χ^2^) test. Student's *T-test* or χ^2^ test was used to analyze differences in clinico-pathological variables between patients with the TERT promoter mutation-positive and negative tumors, respectively. The evaluation of distribution differences of selected variables and alleles of the *TERT* rs2736100 and rs2736098 between patients and healthy controls were done using χ^2^ test. Hardy–Weinberg equilibrium of the genotype distribution among the controls was tested by a goodness-of-fit χ^2^ test. Unconditional univariate and multivariate logistic regression analyses were used to estimate Odds ratios (OR) for risk of HCC or tumors with and without TERT promoter mutation and their 95% confidence interval (CI). All the tests were computed using SigmaStat3.1^®^ software (Systat Software, Inc., Richmond, CA). *P* values of < 0.05 were considered as statistically significant.

## CONCLUSIONS

Our results show that 30% of Chinese HCCs carry TERT promoter mutations and there was a significant difference in genotype distributions of rs2736098 and rs2736100 between patients with wt versus mutant TERT promoter tumors, respectively. The cancer risk genotypes rs2736098_TT and rs2736100_CC are intimately associated with HCC tumors bearing a wt TERT promoter. Based on the present findings, together with experimental and epidemiological data reported by others, we thus suggest that hepatocytes with rs2736098_TT or rs2736100_CC variants are more prone to telomerase activation in oncogenesis, thereby less likely undergoing TERT promoter mutation. Collectively, the germline TERT genetic background may significantly affect the onset of TERT promoter mutations in HCCs, which contributes to better understandings of the mechanism underlying cancer-related telomerase activation.
